# k_L_a based scale-up cultivation of the extremophilic archaeon *Sulfolobus acidocaldarius*: from benchtop to pilot scale

**DOI:** 10.3389/fbioe.2023.1160012

**Published:** 2023-08-07

**Authors:** Kerstin Rastädter, David J. Wurm, Oliver Spadiut, Julian Quehenberger

**Affiliations:** ^1^ Research Division Biochemical Engineering, Institute of Chemical, Environmental and Bioscience Engineering, TU Wien, Vienna, Austria; ^2^ NovoArc GmbH, Vienna, Austria

**Keywords:** bioreactor scale-up, *Sulfolobus acidocaldarius*, k_L_a, mass transfer coefficient, continuous cultivation

## Abstract

The two major scale-up criteria in continuously stirred bioreactors are 1) constant aerated power input per volume (Pg/Vl), and 2) the volumetric O_2_ mass transfer coefficient (kla). However, Pg/Vl is only influenced by the stirrer geometry, stirrer speed, aeration and working volume, while the kla is additionally affected by physiochemical properties of the medium (temperature, pH, salt content, etc.), sparging of gas and also by the bioreactor design. The extremophilic archaeon *Sulfolobus acidocaldarius*, thriving at 75°C and pH 3.0, has the potential for many biotechnological applications. However, previous studies imply that the family Sulfolobaceae might be affected by higher oxygen concentration in the headspace (>26%). Hence, adequate oxygen supply without being toxic has to be ensured throughout the different scales. In this study, the scale-up criteria Pg/Vl and kla were analyzed and compared in a 2 L chemostat cultivation of S. acidocaldarius on a defined growth medium at 75°C and a pH value of 3.0, using two different types of spargers at the same aerated power input. The scale-up criterion kLa, ensuring a high specific growth rate as well as viability, was then used for scaleup to 20 L and 200 L. By maintaining a constant kla comparable dry cell weight, specific growth rate, specific substrate uptake rates and viability were observed between all investigated scales. This procedure harbors the potential for further scale-up to industrial size bioreactors.

## 1 Introduction

Extremophilic archaea, in particular Sulfolobales, harbor great potential as production host for biological compounds such as carotenoids, proteases/lipases and lipids (Quehenberger et al., 2017). Nevertheless, until now no defined industrial process with archaea for biopharmaceutical purposes exists. *Sulfolobus acidocaldarius* is one of the most important model organism for the phylum Crenarchaeota given that a powerful toolbox for genetic engineering is available (Wagner et al., 2012; Peng et al., 2017). Due to its optimal growth at 75°C and pH 3, *S. acidocaldarius* is predestined for the cultivation in continuous mode. Thereby reactor downtime can be reduced and productivity is increased, while contamination with non-target organisms is prevented by the harsh growth conditions. Only a few studies involving Sulfolobales are published where cultivation occurred in a bioreactor scale larger than 3.0 L (Amano et al., 1993; Park and Lee, 1997; Schiraldi et al., 1999; Worthington et al., 2003; Bode et al., 2008) (see Table 1 for comparison). None of these studies operated their cultivation with a defined medium in combination with a chemostat cultivation mode. The biggest scale was a 200 L bioreactor with unspecified stirrer and sparger conditions as well as reactor specifications (Amano et al., 1993; Bode et al., 2008). In general, large-scale archaeal cultivations are impeded by several technical difficulties such as heating and maintaining the temperature over a longer period of time of hyperthermophilic cultures (Worthington et al., 2003) and corrosion resistance of bioreactor materials necessary for the cultivation of alkaliphiles, acidophiles and halophiles (Schiraldi et al., 2002). So far, no scale-up study based on a scale-up criterion rather than trial and error was published with Sulfolobales.

**TABLE 1:** Bioreactor cultivations performed with *Sulfolobus* spp. and related species, *Saccharolobus solfataricus* (formerly known as *Sulfolobus solfataricus*). ^1^OD_540_ divided by correlation factor of 0.586 g/L observed by (Rastädter et al., 2021). n.d.—no data available, n.c.—not calculable.

Strain	Working volume [L]	Cultivation mode	Carbon source	Average biomass space time yield [g_DCW_/L/h]	Fermentation time [h] or dilution rate [h^−1^]	Final biomass titer [g_DCW_/L]	Source
*Sulfolobus acidocaldarius* DG6	9.46	Fed batch, routinely inoculated	Glutamate and starch	0.007	90	∼0.65^1^	Worthington et al. (2003)
*Saccharolobus solfataricus* Gθ	10	Fed batch with microfiltration	Yeast extract, casein hydrolysate acid, glucose	0.113	310	35	Schiraldi et al. (1999)
*Saccharolobus solfataricus* (DSM 1617)	13.8	Constant volume fed- batch	Yeast extract and glucose	0.133	170	22.6	Park and Lee (1997)
*Sulfolobus metallicus*	200	Continuous	Mineral concentrate	n.c.	6 weeks, dilution rate not defined	n.d.	Bode et al. (2008)
*Sulfolobus acidocaldarius* strain 7	200	Batch	Yeast extract, casamino acid and glucose	n.c.	n.d.	n.d.	Amano et al. (1993)
*Sulfolobus acidocaldarius* (DSM 639)	200	Continuous	Glutamate and glucose	0.125	d = 0.03 h^−1^	4.16	this publication

Generally, heterogenous environments due to a lack of mixing and/or mass transfer are the biggest problems in upscaling (Xia et al., 2016). Especially in an aerobic bioreactor, oxygen availability is key and due to the high temperature in bioprocesses with thermophilic organisms, gas solubility is an additional limiting step (Aiba et al., 1984; Deive and Sanromán, 2017). For scale-up different criteria can be used. The most common ones are constant gassed power input per liquid volume (P_g_/V_L_), constant impeller tip speed (ITS) or constant oxygen transfer rate (OTR) as well as constant volumetric O_2_ mass transfer coefficient (k_L_a) (Junker, 2004; Clarke, 2013). The industrial state-of-the-art is either constant P_g_/V_L_ or constant k_L_a (Sweere et al., 1987; Neubauer and Junne, 2016). k_L_a is a function of physical properties of the medium (most importantly the viscosity), as well as geometry of the vessel, type and number of impellers, stirrer speed, type of sparger, gas flowrate, etc. (Garcia-Ochoa and Gomez, 2009; Deive and Sanromán, 2017). The sparger type can have a great impact on the k_L_a value as, for example, a micro sparger disperses the gas into smaller bubble sizes, which alters the residence time in the liquid and specific bubble surface area (Deive and Sanromán, 2017). These parameters not only influence the measured k_L_a value but also have an impact on the microorganism´s physiology throughout the upscale. Cells experience different microenvironments in a large scale reactor caused by aeration and agitation profiles (Hewitt and Nebe-Von-Caron, 2001). These gradients of nutrient concentration and pH throughout the bioreactor as wells as an increase in hydrostatic pressure in large-scale can result in changes in the overall viability (Díaz et al., 2010). Studies in *Escherichia coli* showed the applicability of flow cytometry (FCM) for determining the effect of dissolved oxygen (DO) concentrations, pH and carbon source limitation and agitator speed on the viability (Hewitt et al., 2000; Enfors et al., 2001; Onyeaka et al., 2003). Analogous to these findings in *E. coli* FCM-based viability monitoring can also be used to assess the effect of bioprocess parameters on *S. acidocaldarius* in a timely manner (Rastädter et al., 2022).

Most studies that employed constant power input in their scale-up process additionally maintained the DO above a certain threshold to ensure growth favorable conditions. This is achieved by increasing the oxygen concentration in the inlet gas. The impact of increased oxygen concentrations in the inlet gas during scale-up is generally not investigated in detail, since common production hosts like *E. coli* are very robust towards high oxygen concentrations (Baez and Shiloach, 2013). Nevertheless, iron-sulfur clusters, inherited from the anaerobic ancestors, play a major role in the vulnerability of organisms to oxygen (Imlay, 2006). Small modular Fe-S proteins called ferredoxins have been isolated in *S. acidocaldarius*, grown aerobically (Kerscher et al., 1982; Breton et al., 1995; Duff et al., 1996). These zinc-containing ferredoxins are abundant in the cytoplasm and are involved in electron transport. In aerobic archaea, ferredoxin oxidoreductase is a key Fe-S enzyme in the oxidative TCA cycle (Kerscher et al., 1982; Kerscher and Oesterhelt, 1982; Iwasaki, 2010). Fe-S cluster gets oxidized by superoxide by converting [Fe_4_S_4_]^2+^ to an unstable [Fe_4_S_4_]^3+^ form. Iron gets released and the resulting [Fe_3_S_4_]^1+^—enzyme is inactive (Imlay, 2006). A study with *Saccharolobus solfataricus* (formerly known as *Sulfolobus solfataricus*) confirmed the family’s sensitivity to oxygen by showing that growth was impaired when supplied with higher O_2_ concentrations (>26% headspace O_2_) (Simon et al., 2009).

The present study aims to find an upscaling criterion, constant P_g_/V_L_ or constant k_L_a, for *S. acidocaldarius* to maintain a high specific growth rate and viability considering the organism’s possible sensitivity to higher oxygen concentrations in the gas inflow. This is done by comparing a ring and a micro sparger performed at the same power input yet different k_L_a values in a 2 L bioreactor. Moreover, this criterion is then used for scale-up from benchtop (2 L) to lab (20 L) and to pilot scale (200 L). The effect on viability, dry cell weight, specific growth rate, specific substrate uptake rates and biomass yield throughout the different scales is monitored.

## 2 Materials and methods

### 2.1 Strain and bioreactor cultivations

All cultivations were carried out with the *Sulfolobus acidocaldarius* strain DSM 639, obtained from the German Collection of Microorganism and Cell Cultures (DSMZ, Braunschweig, Germany). 2 L (working volume) chemostat cultivation was executed in a 3 L Biostat A-plus bioreactor (Sartorius, Goettingen, Germany). 20 L (working volume) cultivation was carried out in a 30 L Techfors S bioreactor (Infors HT, Bottmingen, Switzerland). 200 L (working volume) cultivation was performed at the Fraunhofer Center for Chemical and Biotechnological Processes in a 300 L Proreact 20P (Heinrich Frings, Reinbach, Germany). The batch of every bioreactor was started with a Vienna Defined (VD) Medium (Quehenberger et al., 2019) with modified carbon concentrations (2 g/L monosodium glutamate (MSG), 1 g/L D-glucose and 0.25 g/L NZ-amine) and a starting OD_600_ of 0.03–0.08 in a batch volume of 1.5 L, 15 L and 150 L, respectively. An exponential feeding ramp starting with 14.8 g/h, 148 g/h and 1.4 kg/h and a growth rate of 0.035 h^−1^ was applied. After reaching 2, 20, or 200 L cultivation volume the continuous phase was started and a dilution rate of 0.03 h^−1^ was set via the feed rate and kept constant for the time of the experiments. According to a previous conducted study, four theoretical dwell times are needed for reaching a steady state in a chemostat cultivation with this organism (Rastädter et al., 2021). Feed supplied in the fed-batch and chemostat phase contained a 5-times concentrated VD medium with adapted carbon sources and concentrations (9.5 g/L MSG and 4.5 g/L D-glucose). The constant volume of 2 L, 20 L or 200 L was maintained by pumping out cell broth via an immersion tube at a fixed height. During all cultivations the temperature was kept constant at 75°C. The pH value was monitored via an EasyFerm Plus electrode (Hamilton, Reno, NV, United States) and was set to 3.0 by automatic addition of 4.8%–9.6% (v/v) H_2_SO_4_. DO was measured by a Visiferm DO electrode (Hamilton, Reno, NV, United States). In case of the 2 L and 20 L cultivations, CO_2_ and O_2_ concentrations in the off-gas were determined by using a gas analyzing unit (Müller Systems AG, Esslingen, Switzerland) as well as process control and feeding was established using the Lucullus process control system (Securecell, Urdorf, Switzerland). For the 200 L culture, CO_2_ and O_2_ was measured with a SIDOR off-gas analyzing unit (Sick AG, Waldkirch, Germany). GESA (Teuchern, Germany) served as the process control system.

#### 2.1.1 Determination of k_L_a and power inputs

The k_L_a of each scale was determined via measuring the DO in deionized water at 75°C. The DO probe was first calibrated with 100% N_2_ and 100% pressurized air at the given stirrer speed and volume gas flow rate. The dissolved oxygen [%] versus process time [h] curve was then recorded after switching from N_2_ (∼0%) to air until a saturation of ∼ 100% DO was reached. The k_L_a value was then determined with a dynamic oxygen transport equation (Kager, 2022). The stirrer speed and airflow rate in the bioreactors was then set accordingly to reach a k_L_a of ∼ 40 h^−1^. Table 2 shows the set parameters, stirrer speed, airflow rate and used sparger type, for each bioreactor scale. P_g_/V_L_ for each bioreactor was calculated according to Junker (2004). The respective impeller characteristics needed for calculating the power input are shown in Supplementary Table S1.

**TABLE 2:** Summary of bioreactor, sparger configurations used, the respective determined k_L_a and calculated aerated power inputs according to the set parameters. The impeller characteristics necessary for calculating the power input for all different scales are shown in Supplementary Table S1. *Reactor geometry ratio in form of diameter of reactor (D_R_) divided by total volume of the reactor (V_R_).

Bioreactor scale (working volume [L])	Sparger type	Stirrer speed [rpm]	Airflow rate [L/min]	k_L_a [h^−1^]	Aerated power input [kW/m^3^]	Tip speed [m/s]	D_R_/V_R_^*^ratio
2	Micro sparger	355	0.51	38.8	0.25	0.97	40.8
2	Ring sparger	355	0.51	32.6	0.25	0.97	40.8
2	Ring sparger	500	0.51	38.8	0.63	1.36	40.8
20	Ring sparger	285	5.1	40.5	0.25	1.42	7.96
200	Ring sparger	117	37.5	41.1	0.13	1.23	1.57

### 2.2 Sampling

Samples were taken regularly, every 24 h (except for weekends), throughout the chemostat cultivation. The biomass was measured via optical density (OD_600_) as well as via dry cell weight (DCW). Due to different cultivation sites, the determination of viability via flow cytometry (FCM) was not performed during the 200 L cultivation as this took place at the Fraunhofer Center for Chemical-Biotechnological Processes in Leuna, Germany. Concentrations of the substrates, D-glucose and MSG, and metabolites trehalose, were determined in each supernatant.

#### 2.2.1 Setup for 2 L and 20 L scale cultivation

For the 2 L and 20 L cultivation, optical density, OD_600_, was measured at 600 nm using a spectrophotometer, ONDA V-10 PLUS, (XS instruments, Carpi, Italy). Samples were diluted with dH_2_O to stay within the linear range of the photometer. Dry cell weight (DCW) was determined gravimetrically via centrifugation (10,000 x g for 10 min at 4°C) of 1.9 mL of homogenous cell broth in a pre-tared and pre-dried-out 2 mL Greiner Bio-One microcentrifugation tube (Greiner Bio-One, Frickenhausen, Germany). Afterwards, the obtained supernatant was stored at −20°C and later analyzed for its substrate and metabolite composition. The pellet was dried for at least 72 h at 105°C. DCW was then determined gravimetrically in triplicates.

For FCM, 300 µL of cell broth were first centrifuged (10,000 x g, 10 min, 4°C). The remaining pellet was then washed twice and resuspended with 0.2 µm-filtered 10 mM phosphate buffer saline (PBS), pH 5.5. Another centrifugation step (20,000 x g 5 min, 4°C) was performed in between the washing steps. At the final washing step 900 µL of PBS buffer were added to the cell pellet to yield a 1:3 dilution. For staining metabolically active cells, 4.5 µL fluorescein diacetate (FDA, 5 g/L in acetone, Sigma-Aldrich, Burlington, MA, United States) were added to the diluted cell suspension, which was then incubated for 10 min at 37°C in a thermoblock. Afterwards, an additional centrifugation (20,00 x g 5 min, 4°C) and washing step with 900 µL PBS occurred to decrease background noise due to released fluorescein into the supernatant. Subsequently, the sample was further diluted with PBS buffer to yield 1 mL with a dilution of 1:3000. 1 μL of concanvalin A (ConA)—rhodamine (5 g/L, Vector Laboratories, Newark, CA, United States), prior centrifuged to remove any possible protein aggregates, was added to 1 mL diluted FDA-stained cell suspension. The measurements were performed using a Cyflow Cube 8 flow cytometer (Sysmex, Görlitz, Germany). A 488 nm as well as a 532 nm laser were used for excitation. Emission spectra were obtained with the fluorescence channels FL1, 536/40 nm bandpass and FL4, 610/30 nm bandpass filter. Furthermore, forward scatter (FSC) and side scatter (SSC) spectra were recorded. Cells gating and thereby viability determination were done according to Rastädter et al. (2022).

#### 2.2.2 Setup for 200 L scale cultivation

For the OD_600_, the absorbance of the cell broth was measured in a Jenway 7,310 spectrophotometer (Bibby Scientific, Stone, United Kingdom) against a blank of dH_2_O. Samples were diluted with dH_2_O according to the linear range of the photometer. DCW was determined by centrifuging (9,000 x g 10 min, 4°C) 10 mL of homogenously cell broth in pre-tared 50 mL Falcons (Greiner Bio-One, Frickenhausen, Germany). The withdrawn supernatant was stored at −20°C until further analysis of the substrate and metabolite concentrations. The cell pellet was dried for at least 72 h at 95°C and this was done in triplicates.

#### 2.2.3 Substrate and metabolites

D-glucose and trehalose concentration in the clarified cell broth and in the feed was determined with an Aminex HPX-87H column (300 × 7.8 mm, Bio-Rad, Hercules, CA, United States) using a Vanquish Core HPLC system (Thermo Fisher Scientific, Waltham, MA, United States) and a refractive index detector (RefractoMax 520, Thermo Fisher Scientific, Waltham, MA, United States). The mobile phase consisted of 4 mM H_2_SO_4_ with a constant flow rate of 0.6 mL/min. The system was run isocratically at 60 °C. Chromatograms were analyzed using Chromeleon 7.2.6 Chromatography Data System (Thermo Fisher Scientific, Waltham, MA, United States). The concentration of glutamic acid was assessed photometrically with a Cedex Bio HT Analyzer (Roche, Basel, Switzerland). The glutamic acid concentration was then multiplied by 1.15 to obtain the MSG content in the supernatant.

### 2.3 Growth and physiology

All rates, yields and balances, were evaluated at each sampling point and then the mean of the sampling points upon reaching steady state condition in the chemostat was calculated.

*Dilution rate, D* [h^−1^], in the chemostat mode was calculated as the sum of feed and acid addition [L/h] divided by the reactor volume [L].

$$D=\frac{F_{feed}+F_{acid}}{V}$$ (1)

F_feed_ [L/h] feed flow rate

F_acid_ [L/h] acid flow rate

V [L] reactor working volume

*Specific growth rate, µ* [h^−1^], was determined as the difference in DCW between two sampling points divided by the average DCW between the sampling points per hour by taking into account the loss of biomass via the bleed.

$$µ=\frac{∆X+∆V_{bleed}*\bar{x}}{\bar{X}*∆t}$$ (2)

Δt [h] time between two sampling points

ΔX [g] difference of total DCW in broth between the two sampling points

ΔV_bleed_ [L] bleed volume removed between the two sampling points

$\bar{x}$ [g/L] average DCW concentration between sampling points

$\bar{X}$ [g] average biomass in broth between sampling points

*Cell productivity, biomass space time yield, D*⋅*x* [g L^−1^ h^−1^], was calculated as the product of DCW concentration [g/L] and D [h^−1^].

D⋅x=x*D (3)

x [g/L] DCW concentration

D [h^−1^] dilution rate

*Specific substrate uptake rates* for D-glucose, *q*_*Glc*_ [g_Glc_ g(X)^−1^ h^−1^], and L-glutamate, *q*_*MSG*_ [g_MSG_ g(X)^−1^ h^−1^] (shown as MSG per DCW per hour), were calculated for every time span between sampling points as follows:

$$q_{S}=\frac{∆S_{reactor}+S_{in}-S_{out}}{∆t*\bar{X}}$$ (4)

ΔS_reactor_ [g] difference of amount of substrate in broth between two sampling points

S_in_ [g] substrate supplied to bioreactor within the time period

S_out_ [g] substrate discharged via the bleed within the time period

*Specific production rate* of extracellular trehalose *q*_*Tre*_ [g_Tre_ g(X)^−1^ h^−1^] was calculated according to:

$$q_{P}=\frac{∆P_{reactor}+P_{in}-P_{out}}{∆t*\bar{X}}$$ (5)

ΔP_reactor_ [g] difference of amount of product in broth between two sampling points

P_in_ [g] product supplied to bioreactor within the time period

P_out_ [g] product discharged via the bleed within the time period

*Biomass Yield, Y*_*X/S*_ [g(X)/g(S)], was calculated as the quotient of µ [h^−1^] and q_S_ [g(S) g(X)^−1^ h^−1^].

$$Y_{\frac{X}{S}}=\frac{µ}{q_{S}}$$ (6)

*Trehalose yield, Y*_*Tre/S*_ [C-mol_Tre_/C-mol_S_] was calculated as the quotient of q_Tre_ [C-mol_Tre_ g(X)^−1^ h^−1^] and q_S_ [C-mol_S_ g(X)^−1^ h^−1^].

$$Y_{\frac{Tre}{S}}=\frac{q_{Tre}}{q_{S}}$$ (7)

*Carbon evolution rate, CER* [mmol/L/h], was calculated according to:

$$CER=\frac{Q_{g}}{V*Vm}*\left(y_{CO2},out-y_{CO2},in\right)*1000$$ (8)

Q_g_ [L/h] gas inlet flow

V [L] reactor working volume

Vm [L/mol] molar volume of air = 22.414

y_CO2,_ in/out concentration of CO_2_ in inlet and offgas stream

*Oxygen uptake rate, OUR* [mmol/L/h], was calculated as follows:

$$OUR=\frac{Q_{g}}{V*Vm}*\left(y_{O2},out-y_{O2},in\right)*1000$$ (9)

Q_g_ [L/h] gas inlet flow

V [L] reactor working volume

Vm [L/mol] molar volume of air = 22.414

y_O2,_ in/out concentration of O_2_ in inlet and offgas stream

*Respiratory quotient, RQ,* was calculated as the quotient of the carbon evolution rate [mmol L^−1^ h^−1^] and the oxygen uptake rate [mmol L^−1^ h^−1^]. Oxygen uptake rate and CO_2_ evolution rate were calculated by measuring the effluent concentrations of oxygen and CO_2_.

$$RQ=\frac{CER}{OUR}$$ (10)

*Specific carbon dioxide production rate, q*_*CO2*_ [mmol g(X)^−1^ h^−1^], was determined by dividing the carbon evolution rate by the DCW concentration.

$$q_{{CO}_{2}}=\frac{CER}{x}$$ (11)

*Specific oxygen consumption rate, q*_*O2*_ [mmol g(X)^−1^ h^−1^], was determined by dividing the oxygen uptake rate by the DCW concentration.

$$q_{{CO}_{2}}=\frac{OUR}{x}$$ (12)

*CO*_*2*_ *yield, Y*_*CO2/S*_ [C-mol_CO2_/C-mol_S_] was calculated as the quotient of the specific carbon dioxide production rate [C-mol_CO2_ g(X)^−1^ h^−1^] and q_S_ [C-mol_S_ g(X)^−1^ h^−1^].

$$Y_{\frac{{CO}_{2}}{S}}=\frac{q_{CO_{2}}}{q_{S}}$$ (13)

*C-balance* was determined as the sum of Y_X/S_, Y_CO2/S_, and Y_Tre/S_, all in carbon-mol per carbon-mol. A C-balance close to 1.0 implies that all carbon atoms provided via substrate can be accounted for and are recovered either in the biomass (Y_X/S_), in the exhaust gas (Y_CO2/S_) or in metabolites (Y_Tre/S_).

$$C\;balance=Y_{\frac{X}{S}}+Y_{\frac{{CO}_{2}}{S}}+Y_{\frac{Tre}{S}}$$ (14)

*Standard deviation, STD* [%], for each parameter of the reproducibility experiments was calculated as the square root of the variance divided by its mean.

### 2.4 Statistical analysis

Statistical significance for the comparison of the viability in the 2 L scale using different sparger types was done by One-Way ANOVA (OriginLab Corporation, Northampton, MA, United States), followed by a post-hoc analysis using Bonferroni adjustments (significance level of 0.05 divided by compared groups). Therefore, differences were considered to be significant if *p* < 0.0167. The mathematical description of this analysis of variance can be found in the online help section of OriginLab (https://www.originlab.com/doc/en/Origin-Help/OneWayANOVA-Algorithm [Accessed on 23 November 2022].

## 3 Results and discussion

### 3.1 Comparison of power input vs. k_L_a as scale-up criterion

**FIGURE 1:**
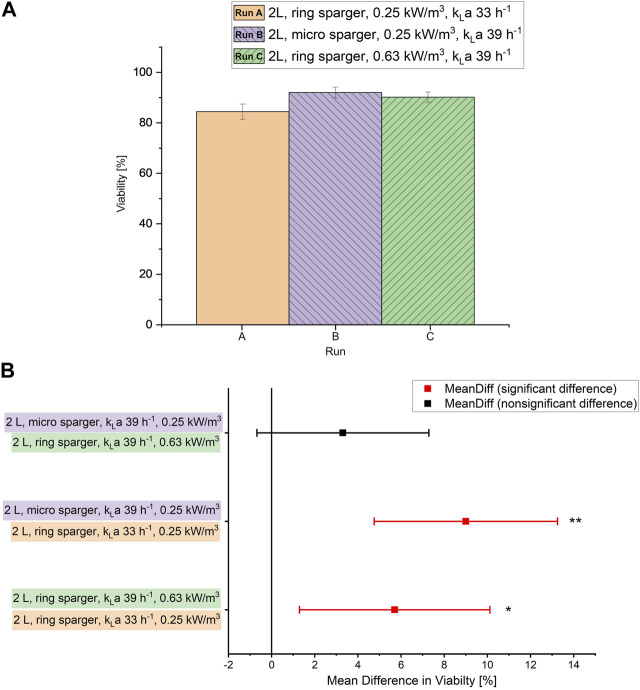
**(A)** Viability [%] measured via flow cytometry during the chemostat phase at each sparger type and k_L_a value in the 2 L scale. Error bars indicate the deviation between the various sampling points after reaching steady state in the chemostat phase. **(B)** Mean difference in viability, significance according to ANOVA: single factor and post-hoc analysis with Bonferroni-adjustment calculated in OriginPro 2019 (OriginLab Corporation, Northampton, MA, United States). **p < 0.01; * *p* < 0.05.

To determine a scale-up criterion for bioprocesses with *S. acidocaldarius* that allows for a high specific growth rate and viability, 2 different sparger types (micro and ring sparger) were tested in the 2 L scale at the same power input but different k_L_a values (Table 2). In the setup with the ring sparger (2 L, aerated power input 0.25 kW/m^3^), limiting dissolved oxygen (<20%, Supplementary Figure S2A) was observed due to a low k_L_a value of 32.6 h^−1^. Hence, additional oxygen had to be supplied via the gas inflow. This caused a drop in the viability compared to the setup equipped with micro sparger (2 L, aerated power input 0.25 kW/m^3^) where no additional oxygen had to be supplied via the inflow due to a higher k_L_a of 38.8 h^−1^ (Figure 1). The viability in the setup with ring sparger increased by almost 6% when the k_L_a value was set to 38.8 h^−1^ by increasing the stirrer speed (2 L, aerated power input 0.63 kW/m^3^) and omitting additional supply of oxygen via the gas inflow. No significant difference in viability was observed between micro and ring sparger setup when the same k_L_a values were applied (Figure 1). Furthermore, no difference was observed in the specific uptake rates of carbon sources, the growth rates, the biomass production and in the OD_600_ values (shown in the Supplementary Material; Supplementary Figures S1A,B, S2A). This suggests that the low viability in the culture might be solely caused by the extra oxygen supply confirming the organism’s oxygen sensitivity demonstrated in the related species *Sa. solfataricus* (Simon et al., 2009).

Hence, the scale-up was based on constant k_L_a rather than specific aerated power input to ensure enough dissolved oxygen supply without having to supply extra O_2_. The ring sparger design was used for further scale-up as it is the industry standard and generally available in all bioreactor scales.

### 3.2 k_L_a based scale-up

In the benchtop scale of 2 L, it was shown that a k_L_a of at least 38.8 h^−1^ was necessary for sufficient dissolved oxygen supply (>20%), therefore the scale-up to 20 L and 200 L scale was then based on a constant k_L_a of ∼ 40 h^−1^. The k_L_a was set by adjusting the stirrer speed and airflow rate (Table 2). In the 20 L and 200 L scale a slight overhead pressure of 0.1–0.3 bar was applied but was kept constant throughout the chemostat phase. Table 2 shows the process parameters of each bioreactor scale and sparger type. The k_L_a measurements showed a mean standard deviation of ±1.17 [h^−1^], hence ≤ 3% for all estimated k_L_a values.

#### 3.2.1 Comparison of physiology, growth, and viability during scale-up

**FIGURE 2:**
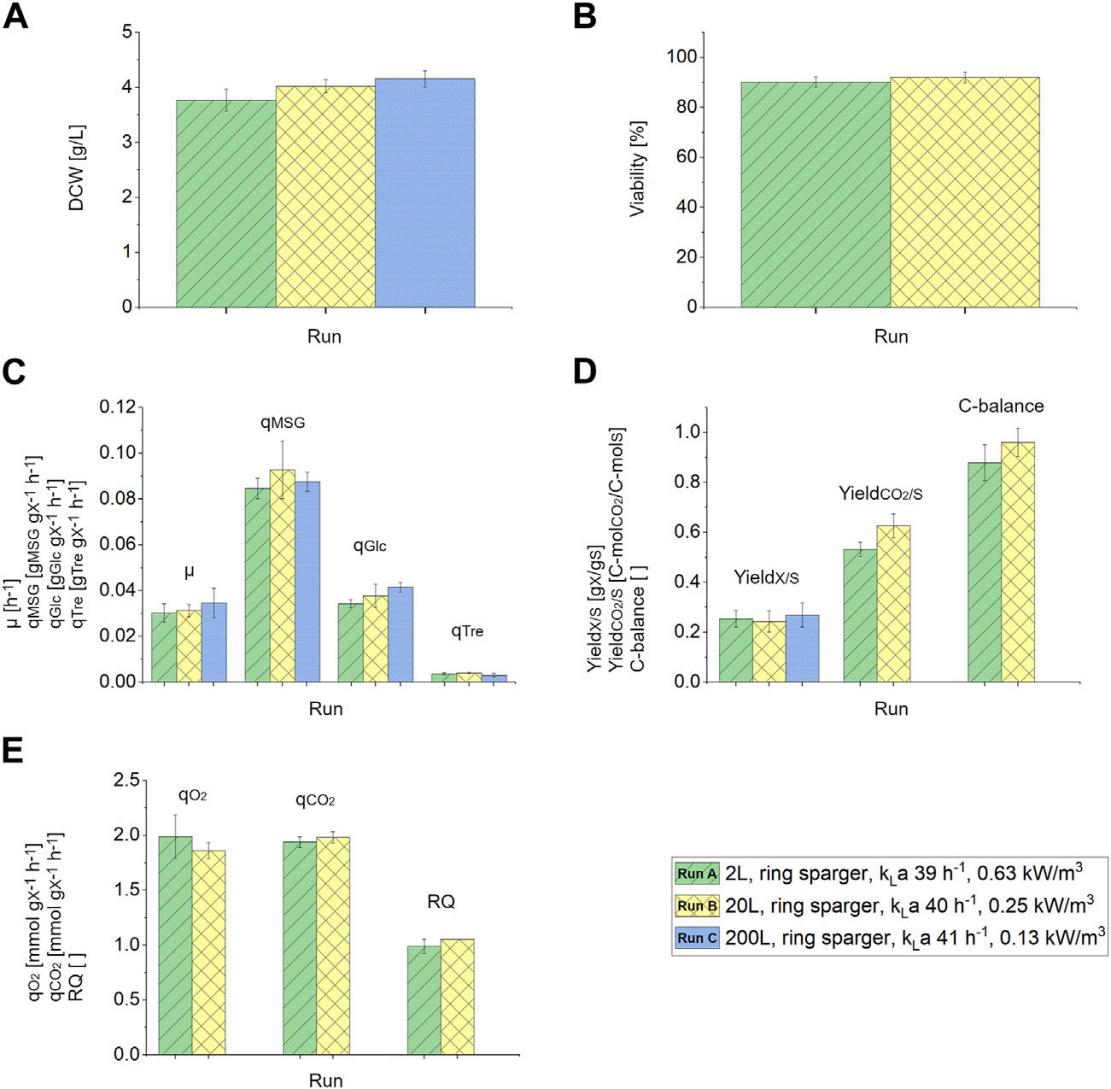
**(A)** Dry cell weight (DCW) [g/L] in response to the different scales 2 L (ring sparger, k_L_a 39 h^−1^, 0.63 kW/m^3^), 20 L (ring sparger, k_L_a 40 h^−1^, 0.25 kW/m^3^) and 200 L (ring sparger, k_L_a 41 h^−1^, 0.13 kW/m^3^). **(B)** Viability [%] measured via FCM during the 2 L and 20 L chemostat phase. **(C)** Growth rate, µ [h^−1^], specific substrate uptake rates, q_MSG_ and q_Glc_ [g_S_/g_X_/h], specific formation rate of trehalose, q_Tre_ [g_p_/g_x_/h], **(D)** biomass yield, Y_X/S_ [g_X_/g_S_], CO_2_ yield, Y_CO2/S_ [C-mol_CO2_/C-mol_S_] and C-balance in response to different bioreactor scales (2 L, 20 L and 200 L) with constant k_L_a values. **(E)** Specific oxygen consumption rate, q_O2_ [mmol_O2_/g_X_/h], specific carbon dioxide production rate, q_CO2_ [mmol_CO2_/g_X_/h] and respiratory quotient (RQ) with reference to different bioreactor scales (2 L and 20 L). Error bars indicate the deviation between the various sampling points after reaching steady state in the chemostat phase.

The measured DCW slightly increased from 3.77 to 4.16 g/L during the scale-up, respectively (Figure 2A). The viability was not influenced by the scale-up from 2 L to 20 L and was around 91% (Figure 2B). Viability measurements were not performed during the 200 L scale. The growth rate, µ, the specific uptake rates of the two carbon sources, q_MSG_ and q_Glc_, the specific production rate of trehalose, q_Tre_ (Figure 2C), as well as the biomass yield, Y_X/S_, CO_2_ yield, and C-balance (Figure 2D) were comparable throughout the scale-up. Additionally, the specific oxygen consumption rate, q_O2_, specific carbon dioxide production rate, q_CO2_, and the respiratory quotient were comparable between the 2 L and 20 L scale (Figure 2E). Due to faulty CO_2_ and O_2_ measurements in the off-gas of the 200 L reactor CO_2_ yield, C-balance, q_O2_, q_CO2_ and RQ could not be calculated for this scale. All other physiological rates were comparable throughout the scales, therefore also for the 200 L scale no metabolite formation in addition to trehalose was assumed.

Table 1 shows the comparison of already published larger-scale bioreactor cultivation performed with Sulfolobales and this 200 L process with *S. acidocaldarius* in terms of working volume, cultivation mode, used carbon source, biomass space time yield, fermentation time and final biomass titer. Higher cell densities of 35 and 22.6 g/L in *Sa. solfataricus* in contrast to 4.16 g/L observed in this study in *S. acidocaldarius* were achieved by employing fed-batch with microfiltration (Schiraldi et al., 1999) or by using complex media in a fed-batch cultivation (Park and Lee, 1997). Unfortunately, biomass concentration data for the other 200 L cultivation were not published (Amano et al., 1993; Bode et al., 2008). While in the present study the observed biomass titer is substantially lower compared to previous studies (Table 1), due to the applied continuous cultivation mode and therefore continuous harvest of biomass, the biomass space time yield is comparable to the fed-batch modes previously performed with *Sa. solfataricus* and almost 18 times higher than observed in a *S. acidocaldarius* lab-scale cultivation (Worthington et al., 2003).

## 4 Conclusion

In this work, a bioprocess scale-up on the basis of constant volumetric oxygen mass transfer coefficient, k_L_a, from benchtop (2 L) to lab scale (20 L) and pilot scale (200 L) was performed. A k_L_a based approach was chosen over the P_g_/V_L_-based scale-up, since experiments in the benchtop scale with different sparger types showed a potential sensitivity of cells to higher oxygen concentrations in the headspace which had to be supplied to compensate decreasing dissolved oxygen levels in the broth. The conducted scale-up to 20 L and 200 L showed similar results in the cultivation regarding the physiology, growth, and viability (not performed at 200 L). Altogether, it was shown that scale-up of the fermentation process was possible with *S. acidocaldarius* in a continuous mode with comparable biomass space time yields throughout the different scales. With this k_L_a-based scale-up approach another hurdle on the path towards an industrial scale (2000 L) process with this untapped archaeal resource was tackled.

## Data Availability

The raw data supporting the conclusion of this article will be made available by the authors, without undue reservation.

## References

[B1] Aiba S. Koizumi J. Ru J. S. Mukhopadhyay S. N. (1984). The effect of temperature on KL a in thermophilic cultivation of *Bacillus stearothermophilus* . Biotechnol. Bioeng. 26, 1136–1138. 10.1002/bit.260260921 18553540

[B2] Amano T. Wakagi T. Oshima T. (1993). An ecto-enzyme from *Sulfolobus acidocaldarius* strain 7 which catalyzes hydrolysis of inorganic pyrophosphate, ATP, and ADP: Purification and characterization. J. Biochem. 114, 329–333. 10.1093/oxfordjournals.jbchem.a124176 8282721

[B3] Baez A. Shiloach J. (2013). *Escherichia coli* avoids high dissolved oxygen stress by activation of SoxRS and manganese-superoxide dismutase. Microb. Cell Fact. 12, 23. 10.1186/1475-2859-12-23 23497217 PMC3605374

[B4] Bode M. L. Buddoo S. R. Minnaar S. H. du Plessis C. A. (2008). Extraction, isolation and NMR data of the tetraether lipid calditoglycerocaldarchaeol (GDNT) from *Sulfolobus metallicus* harvested from a bioleaching reactor. Chem. Phys. Lipids 154, 94–104. 10.1016/j.chemphyslip.2008.02.005 18339312

[B5] Breton J. L. Duff J. L. C. Butt J. N. Armstrong F. A. George S. J. Pétillot Y. (1995). Identification of the iron-sulfur clusters in a ferredoxin from the archaeon *Sulfolobus acidocaldarius* . Eur. J. Biochem. 233, 937–946. 10.1111/j.1432-1033.1995.937_3.x 8521862

[B6] Clarke K. G. (2013). “9 - bioprocess scale up,” in Bioprocess engineering. Editor Clarke K. G. (Sawstone: Woodhead Publishing), 171–188. 10.1533/9781782421689.171

[B7] Deive F. J. Sanromán M. Á. (2017). “14 - bioreactor development for the cultivation of extremophilic microorganisms,” in Current developments in Biotechnology and bioengineering. Editors Larroche C. Sanromán M. Á. Du G. Pandey A. (Amsterdam: Elsevier), 403–432. 10.1016/B978-0-444-63663-8.00014-8

[B8] Díaz M. Herrero M. García L. A. Quirós C. (2010). Application of flow cytometry to industrial microbial bioprocesses. Biochem. Eng. J. 48, 385–407. 10.1016/j.bej.2009.07.013

[B9] Duff J. L. C. Breton J. L. J. Butt J. N. Armstrong F. A. Thomson A. J. (1996). Novel redox chemistry of [3Fe−4S] clusters: Electrochemical characterization of the all-Fe(II) form of the [3Fe−4S] cluster generated reversibly in various proteins and its spectroscopic investigation in *Sulfolobus acidocaldarius* ferredoxin. J. Am. Chem. Soc. 118, 8593–8603. 10.1021/ja961465l

[B10] Enfors S. O. Jahic M. Rozkov A. Xu B. Hecker M. Jürgen B. (2001). Physiological responses to mixing in large scale bioreactors. J. Biotechnol. 85, 175–185. 10.1016/S0168-1656(00)00365-5 11165362

[B11] Garcia-Ochoa F. Gomez E. (2009). Bioreactor scale-up and oxygen transfer rate in microbial processes: An overview. Biotechnol. Adv. 27, 153–176. 10.1016/j.biotechadv.2008.10.006 19041387

[B12] Hewitt C. J. Nebe-Von Caron G. Axelsson B. McFarlane C. M. Nienow A. W. (2000). Studies related to the scale-up of high-cell-density *E. coli* fed-batch fermentations using multiparameter flow cytometry: Effect of a changing microenvironment with respect to glucose and dissolved oxygen concentration. Biotechnol. Bioeng. 70, 381–390. 10.1002/1097-0290(20001120)70:4<381::aid-bit3>3.0.co;2-0 11005920

[B13] Hewitt C. J. Nebe-Von-Caron G. (2001). An industrial application of multiparameter flow cytometry: Assessment of cell physiological state and its application to the study of microbial fermentations. Cytometry 44, 179–187. 10.1002/1097-0320(20010701)44:3<179::AID-CYTO1110>3.0.CO;2-D 11429768

[B14] Imlay J. A. (2006). Iron-sulphur clusters and the problem with oxygen. Mol. Microbiol. 59, 1073–1082. 10.1111/j.1365-2958.2006.05028.x 16430685

[B15] Iwasaki T. (2010). Iron-sulfur world in aerobic and hyperthermoacidophilic archaea *Sulfolobus* . Archaea 2010, 1–14. 10.1155/2010/842639 PMC2946596 20885930

[B16] Junker B. H. (2004). Scale-up methodologies for *Escherichia coli* and yeast fermentation processes. J. Biosci. Bioeng. 97, 347–364. 10.1016/s1389-1723(04)70218-2 16233642

[B17] Kager J. (2022). kLa determination with dynamic oxygen transport equation. Available at: https://gitlab.com/juliankager/kla_determination (Accessed September 22, 2022).

[B18] Kerscher L. Nowitzki S. Oesterhelt D. (1982). Thermoacidophilic archaebacteria contain bacterial-type ferredoxins acting as electron acceptors of 2-oxoacid: Ferredoxin oxidoreductases. Eur. J. Biochem. 128, 223–230. 10.1111/j.1432-1033.1982.tb06955.x 6816594

[B19] Kerscher L. Oesterhelt D. (1982). Pyruvate: Ferredoxin oxidoreductase — New findings on an ancient enzyme. Trends Biochem. Sci. 7, 371–374. 10.1016/0968-0004(82)90118-9

[B20] Neubauer P. Junne S. (2016). “Scale-up and scale-down methodologies for bioreactors,” in Bioreactors (Weinheim: Wiley-VCH), 323–354. 10.1002/9783527683369.ch11

[B21] Onyeaka H. Nienow A. W. Hewitt C. J. (2003). Further studies related to the scale-up of high cell density *Escherichia coli* fed-batch fermentations. Biotechnol. Bioeng. 84, 474–484. 10.1002/bit.10805 14574706

[B22] Park C. B. Lee S. B. (1997). Constant-volume fed-batch operation for high density cultivation of hyperthermophilic aerobes. Biotechnol. Tech. 11, 277–281. 10.1023/A:1018402908595

[B23] Peng N. Han W. Li Y. Liang Y. She Q. (2017). Genetic technologies for extremely thermophilic microorganisms of *Sulfolobus*, the only genetically tractable genus of crenarchaea. Sci. China Life Sci. 60, 370–385. 10.1007/s11427-016-0355-8 28251462

[B24] Quehenberger J. Albersmeier A. Glatzel H. Hackl M. Kalinowski J. Spadiut O. (2019). A defined cultivation medium for *Sulfolobus acidocaldarius* . J. Biotechnol. 301, 56–67. 10.1016/j.jbiotec.2019.04.028 31153897

[B25] Quehenberger J. Shen L. Albers S. V. Siebers B. Spadiut O. (2017). *Sulfolobus* - a potential key organism in future Biotechnology. Front. Microbiol. 8, 2474. 10.3389/fmicb.2017.02474 29312184 PMC5733018

[B26] Rastädter K. Tramontano A. Wurm D. J. Spadiut O. Quehenberger J. (2022). Flow cytometry-based viability staining: An at-line tool for bioprocess monitoring of *Sulfolobus acidocaldarius* . Amb. Express 12, 107. 10.1186/s13568-022-01447-1 35947320 PMC9365904

[B27] Rastädter K. Wurm D. J. Spadiut O. Quehenberger J. (2021). Physiological characterization of *Sulfolobus acidocaldarius* in a controlled bioreactor environment. Int. J. Environ. Res. Public Health 18, 5532. 10.3390/ijerph18115532 34064179 PMC8196767

[B28] Schiraldi C. Giuliano M. De Rosa M. (2002). Perspectives on biotechnological applications of archaea. Archaea 1, 75–86. 10.1155/2002/436561 15803645 PMC2685559

[B29] Schiraldi C. Marulli F. Di Lernia I. Martino A. De Rosa M. (1999). A microfiltration bioreactor to achieve high cell density in *Sulfolobus solfataricus* fermentation. Extremophiles 3, 199–204. 10.1007/s007920050117 10484176

[B30] Simon G. Walther J. Zabeti N. Combet-Blanc Y. Auria R. Van Der Oost J. (2009). Effect of O2 concentrations on *Sulfolobus solfataricus* P2. FEMS Microbiol. Lett. 299, 255–260. 10.1111/j.1574-6968.2009.01759.x 19735462

[B31] Sweere A. P. J. Luyben K. Ch. A. M. Kossen N. W. F. (1987). Regime analysis and scale-down: Tools to investigate the performance of bioreactors. Enzyme Microb. Technol. 9, 386–398. 10.1016/0141-0229(87)90133-5

[B32] Wagner M. van Wolferen M. Wagner A. Lassak K. Meyer B. H. Reimann J. (2012). Versatile genetic tool box for the crenarchaeote *Sulfolobus acidocaldarius* . Front. Microbiol. 3, 214. 10.3389/fmicb.2012.00214 22707949 PMC3374326

[B33] Worthington P. Blum P. Perez-Pomares F. Elthon T. (2003). Large-scale cultivation of acidophilic hyperthermophiles for recovery of secreted proteins. Appl. Environ. Microbiol. 69, 252–257. 10.1128/aem.69.1.252-257.2003 12514002 PMC152466

[B34] Xia J. Wang G. Lin J. Wang Y. Chu J. Zhuang Y. (2016). “Advances and practices of bioprocess scale-up,” in Bioreactor engineering research and industrial applications II. Editors Bao J. Ye Q. Zhong J. J. (Berlin, Heidelberg: Springer), 137–151. 10.1007/10_2014_293 25636486

