# Supplementary material for: kLa based scale-up cultivation of the extremophilic archaeon Sulfolobus acidocaldarius: from benchtop to pilot scale
Source: Front Bioeng Biotechnol. 2023 Aug 7;11:1160012. doi: 10.3389/fbioe.2023.1160012 (PMC10441222; doi:10.3389/fbioe.2023.1160012)
Supplement: Supplementary file 1 [file DataSheet1.docx]

Supplementary materials

**TABLE S1.** Summary of impeller characteristics for all different used bioreactor scales.

| bioreactor scale (working volume) | Impeller types | Number of blades | Submerged impellers | Impeller diameter  [mm] | Impeller blade height [mm] |
| --- | --- | --- | --- | --- | --- |
| 2 L | Rushton | 6 | 2 | 52 | 10 |
| 20 L | Rushton | 6 | 3 | 95 | 13.22 |
| 200 L | Rushton | 6 | 4 | 200 | 40 |


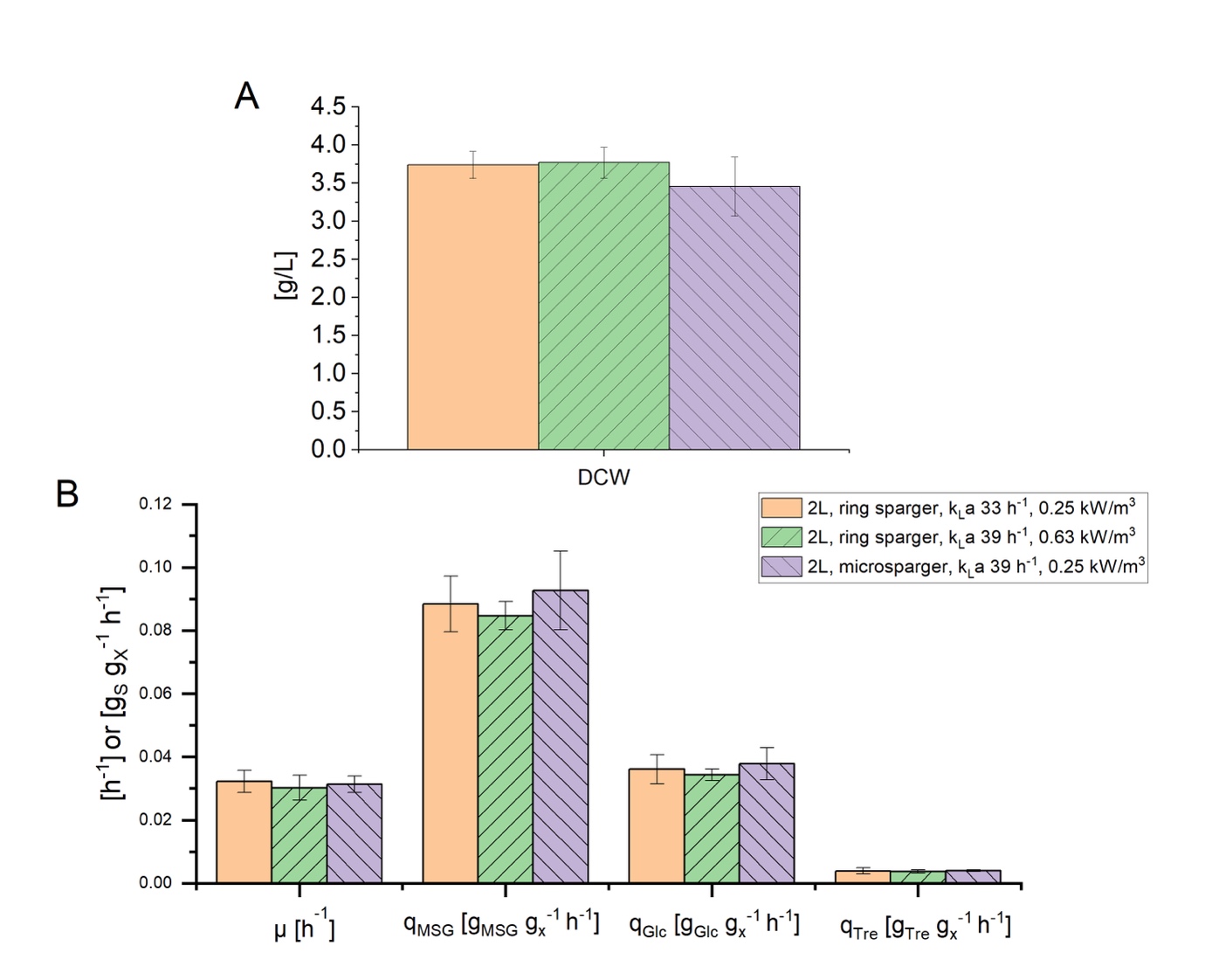


**Figure S1 (A)** dry cell weight (DCW) [g/L] in response to the different sparger types, ring and micro sparger, at different k_L_a values in the 2 L scale. **(B)** growth rate, µ [h^-1^], specific substrate uptake rates, q_MSG_ and q_Glc_ [g_S_/g_X_/h], and specific formation rate of trehalose, q_Tre_ [g_p_/g_x_/h]. Error bars indicate the deviation between the various sampling points after reaching steady state in the chemostat phase.


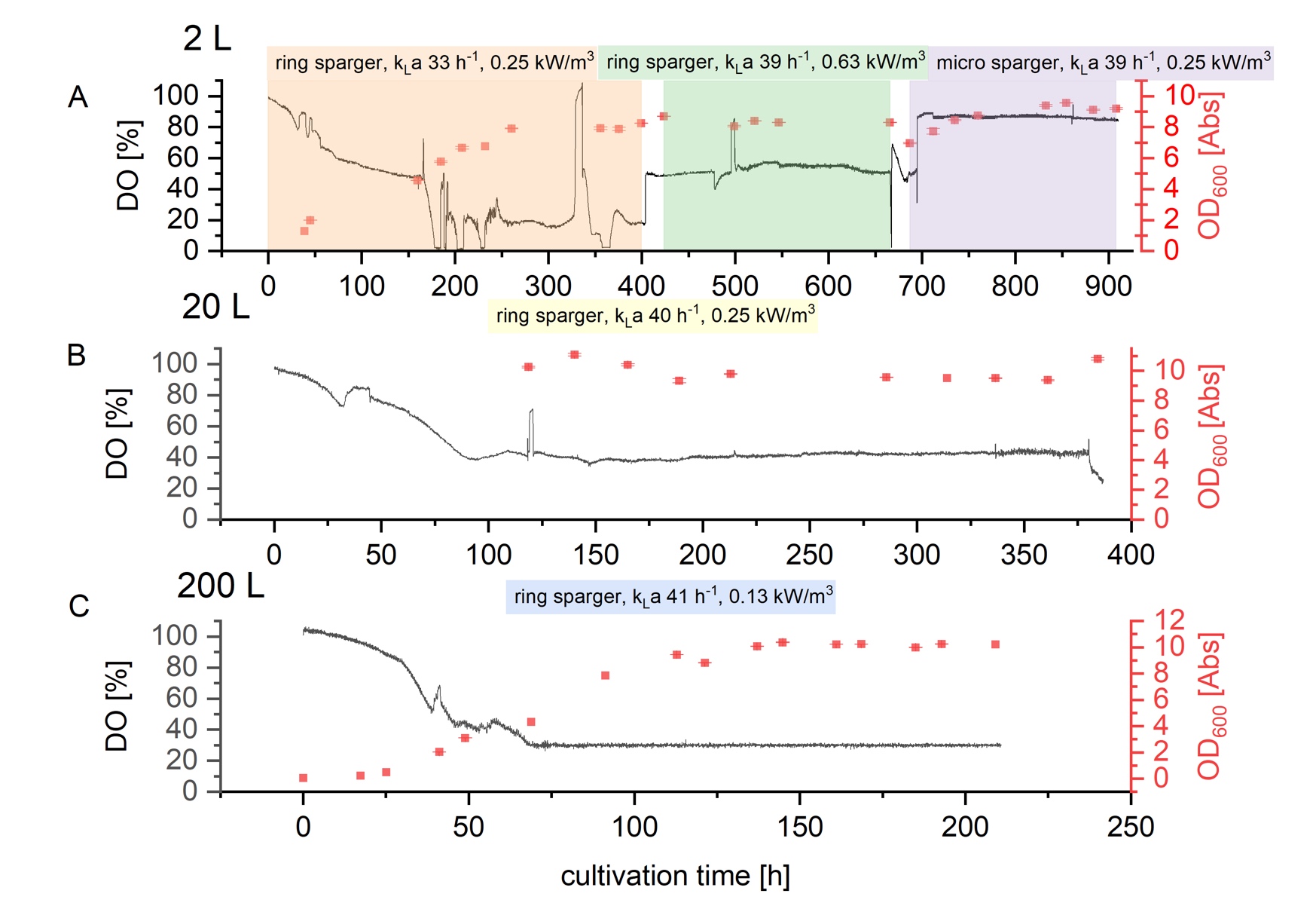


**Figure S2** Course of dissolved oxygen [%] and OD_600_ [Abs] in **(A)** 2 L continuous cultivation in response to the different sparger types, ring and micro sparger, at different k_L_a values; in **(B)** 20 L continuous cultivation; **(C)** 200 L cultivation. Note that in case of the 2 L cultivation as the sparger type as well as the stirrer speed was changed during the cultivation the calibration of the dissolved oxygen is hence after the first condition not anymore accurate.
